# Aminoethyl substitution enhances the self-assembly properties of an aminocellulose as a potential archaeological wood consolidant

**DOI:** 10.1007/s00249-020-01451-y

**Published:** 2020-08-25

**Authors:** Jennifer M. K. Wakefield, Robert Hampe, Richard B. Gillis, Agnes Sitterli, Gary G. Adams, Hartmut Kutzke, Thomas Heinze, Stephen E. Harding

**Affiliations:** 1 National Centre for Macromolecular Hydrodynamics (NCMH), School of Biosciences, University of Nottingham, Sutton Bonington, LE12 5RD UK; 2 School of Chemistry, University of Nottingham, University Park, Nottingham, NG7 2RD UK; 3 Queen’s Medical Centre, School of Health Sciences, University of Nottingham, Nottingham, NG7 2HA UK; 4 Institut für Organische Chemie und Makromolekulare Chemie, Kompetenzzentrum Polysaccharidforschung, Friedrich-Schiller-Universität Jena, Humboldtstrasse 10, 07743 Jena, Germany; 5 Museum of Cultural History, University of Oslo, Postbox 6762, St. Olavs plass, 0130 Oslo, Norway

**Keywords:** Sedimentation velocity, Sedimentation equilibrium, MULTISIG, Hydroxyethyl aminocellulose, Self-association

## Abstract

The 6-deoxy-6-aminocelluloses—or “aminocelluloses”—are a class of synthetic natural cellulose derivatives which are mostly aqueous soluble and have excellent film-forming properties. Recent studies have connected these properties at the molecular level with protein-like self-associative behaviour for a range of aminocelluloses including a 6-deoxy-6-(ω-aminoethyl) aminocellulose AEA-1 with the association being a two-stage process—a reversible oligomerisation followed by further (semi-reversible) aggregation into larger structures. Here, we synthesise and compare a new 6-deoxy-6-(ω-aminoethyl) aminocellulose AEA-1′ with different degree of substitution with one with further alkyl derivatisation, namely 6-deoxy-6-(ω-hydroxyethyl) aminocellulose HEA-1′. As with AEA-1, sedimentation velocity and sedimentation equilibrium in the analytical ultracentrifuge still show a two-stage process for both AEA-1′ and HEA-1′, with the latter giving higher molar masses. The consequences of these properties for use as consolidants for archaeological wood are considered.

## Introduction

Cellulose—a ubiquitous natural polymer consisting of glucose units—possesses a variety of interests. The hydroxyl groups present in the polysaccharide backbone make the chemical modification and thus the design of new functional bio-based material accessible (Heinze and Liebert 2012). One possibility for chemical modification is the conversion of the primary hydroxyl group of the glucose unit with *p*-toluenesulfonyl chloride to obtain cellulose tosylate (Rahn et al. 1996). 6-deoxy-6-aminocellulose (usually called aminocellulose) can be prepared by nucleophilic displacement (S_N_2 mechanism) of tosylate with amines (Heinze et al. 2016). The conversion of cellulose tosylate with di- or oligo-alkylamines can be applied to obtain water-soluble cellulose derivatives (6-deoxy-6-(ω-aminoalkyl) aminocellulose) with terminal amino groups of the substituent. Aminocelluloses overall have already a variety of potential applications mainly in the biomedical field (Reis et al. 2008; Petersen and Gatenholm 2011; Ulery et al. 2011; Croisier and Jérôme 2013; Kumbar et al. 2014; Heinze et al. 2016; McHale et al. 2016). These substances have previously been investigated as a coating for glass to investigate if they could be used to coat biomedical equipment such as implants with a biocompatible/biofunctional film and for antimicrobial activity, and positive results have been found (Jung and Berlin 2005; Jung et al. 2007; Roemhild et al. 2013). Another potential role is in the use of catheter-like devices, where the aminocellulose can prevent biofilm formation and hence potentially prevent infection (Francesko et al. 2016). These rely on layers of aminocellulose and hyaluronic acid: inherent self-association properties of aminocellulose could affect this and impact how easily this coating could be removed from devices. Furthermore, the bactericidal activity of aminocellulose has been shown on aminofunctionalised cellulose nanofibers prepared by electrospinning; of 6‐deoxy‐6‐(trisamino­ethyl) aminocellulose bearing two terminal amino groups per substituent. In a similar way, 6-deoxy-6-(ω-aminoalkyl) aminocellulose can be used as a coating for wound dressings (Roemhild et al. 2013) self-association affecting the thickness of layers and how readily they can be re-dissolved after the cellulosic surface has been coated and dried out could be very important to this application. Other applications have been described (see, for example, Jedvert et al. 2017).

There is another potentially hugely important application. Currently used materials to preserve archaeological wood have a number of drawbacks: there is a tendency to ‘plastify’ the objects, they do not allow re-treatment which may become necessary after a certain period of time, they lose stability over time, and they do not counteract threats to the wood from the likes of high acidity and presence of metal ions. Therefore, intense research efforts are going on to design a new generation of wood consolidants. The great success of developing biomimetic and bio-inspired materials in other fields than conservation—such for the above-mentioned biomedical applications—gave reason to focus on biopolymers and their derivatives (Christensen et al. 2012; Walsh et al. 2017; Wakefield et al. 2018). Early attempts to use wood components like cellulose to consolidate archaeological wood were not completely satisfying (Cipriani et al. 2010). As is well known in the research on biomimetic materials, the natural model cannot be 1:1 transferred into a technical application (Fratzl 2007). Therefore, modifications of natural polymers are synthesised and tested for conservation purposes. This allows us also to tailor the molecules for specific applications and conservation challenges.

The biocompatibility and the adsorption of aminocellulose onto cellulosic surfaces make them promising materials for wood coating and conservation. An important prerequisite is that the molar mass is low enough to penetrate the cells and the aminocellulose adheres to the remaining wood fibre (or other consolidants being used). A second is the ability to cure or network to give a strong stable structure once inside the wood—any self-assembly property would help in that regard. This makes aminocellulose a strong candidate—it may strengthen the wood by providing a coating around each cell, therefore the wood as a whole. The amino groups provide solubility in water to allow treatment and may also prevent bacterial and fungal growth. They may also chelate metal ions in wooden artefacts which contain iron components or iron migrated into the wood during burial: the presence of iron is well known to contribute significantly to the degradation of wood. Finally, the amino groups may provide a small alkaline reserve to help with the prevention of future acid build-up and degradation. This is very important in terms of the Oseberg artefacts (see also the previous article by Wakefield et al 2018) which are currently highly acidic, incredibly fragile and, therefore, require re-conservation (Braovac et al. 2018). Established consolidants have some disadvantages and aminocellulose may prove a good alternative (Almkvist and Persson 2008).

**Fig. 1:**
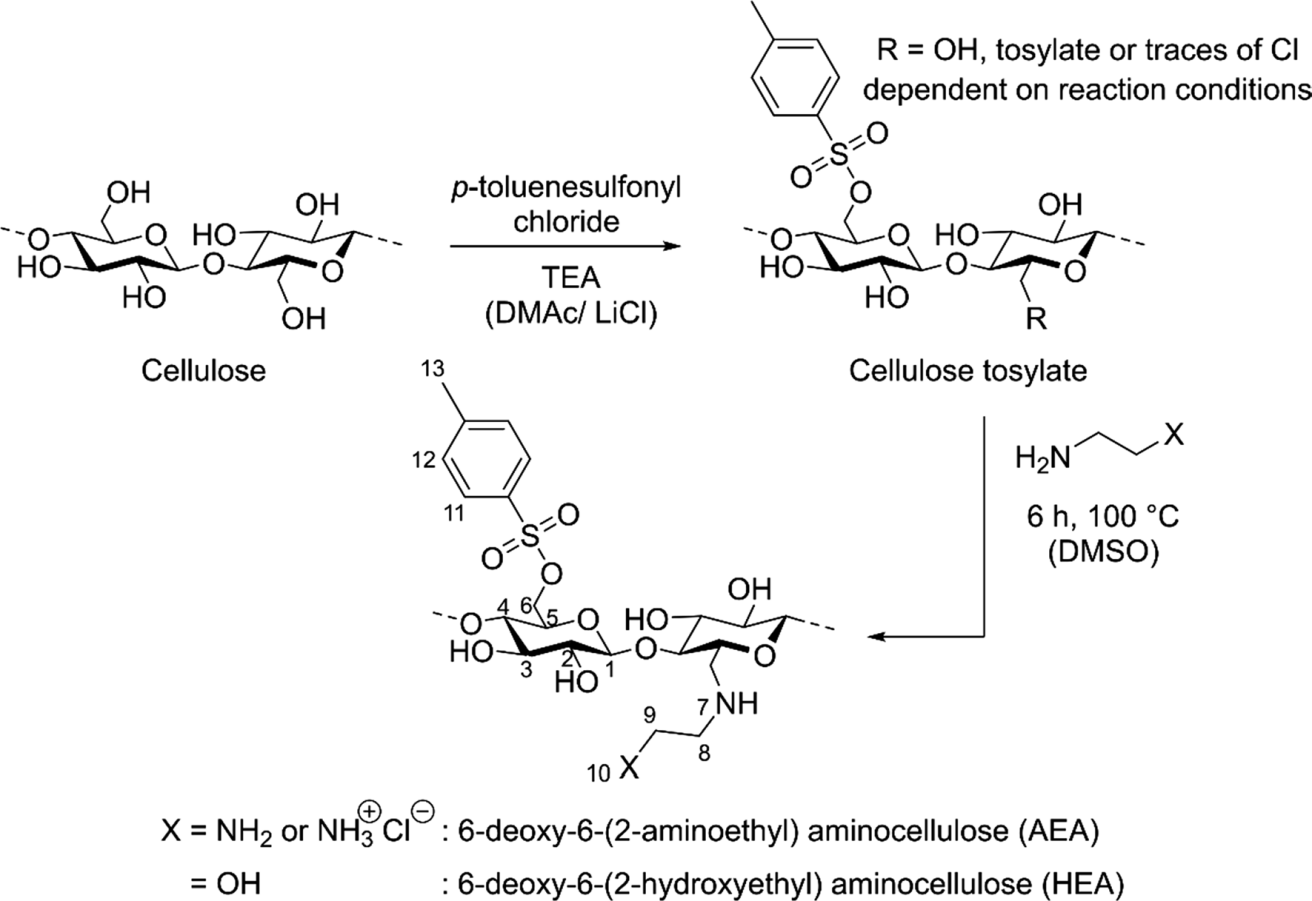
Synthesis diagram for nucleophilic displacement (S_N_2) of tosylate group with an amino- or hydroxyl-terminated ethylamine to obtain the AEA (X = NH_2_) and HEA (X = OH) classes of aminocelluloses

As a first step in gaining a deeper insight into the structure–property relationships of aminocelluloses for potential application to the field of wood conservation, we investigate the influence of altering the degree of substitution (DS) and the terminating end group of the alkylamine used for nucleophilic displacement of tosylate and residual tosylate/6-deoxy-6-chloro groups (Fig. 1) on the self- assembly process. We use a similar approach for monitoring the self-assembly process as used previously for the characterisation of other aminocelluloses, namely sedimentation velocity to examine nature and the extent of the association state of these substances, and sedimentation equilibrium to assess the state of reversibility of these processes and estimate the monomer molar mass (Heinze et al. 2011; Nikolajski et al. 2014). These analytical ultracentrifuge methods are particularly suited for the analysis of these self-associating systems as they combine a matrix-free separation with an absolute analytical facility [in the case of sedimentation equilibrium not requiring assumptions concerning conformation of the polymeric species (Harding et al. 2015)]. Another advantage, compared with classically used gel permeation chromatography (GPC) approaches, is that sedimentation equilibrium is absolute and does not require comparison with standard polymers of known molar mass with a similar conformation: GPC works well when a polymer has very similar properties to the standards, but when these properties deviate, the error can be large (see, for example, Morris et al. 2009). Sedimentation velocity and sedimentation equilibrium are also well-known sister analytical ultracentrifuge techniques not only for finding the sedimentation coefficient and molar mass but also to investigate self-association in macromolecular solutions, popularly applied to proteins and more recently aminocelluloses which were shown to have remarkable protein-like properties (Heinze et al. 2011; Nikolajski et al. 2014).

## Materials and methods

### Aminocelluloses

The aminocellulose 6-deoxy-6-(2-aminoethyl) aminocellulose (AEA-1′; Fig. 1) was synthesised as described previously for AEA-1 (DS_amine_ = 0.83, DS_tos_ = 0.20) (Nikolajski et al. 2014) but with a different degree of substitution: DS_amine_ = 0.59 (59% of neutral form: DS = 0.35 and 41% of cationic form: DS = 0.24, as determined by elemental analysis of the N and Cl content, respectively) and there is no residual tosylate (DS_tos_). The hydroxyl derivative 6-deoxy-6-(2-hydroxyethyl) aminocellulose (HEA-1′) was synthesised in a similar manner to AEA-1′ from cellulose tosylate but by reacting ethanolamine (Fig. 1). For HEA-1′ DS_amine_ = 0.69 and DS_tos_ = 0.09, the chloride found in sample HEA-1′ by elemental analysis is considered a counter ion of the secondary amine.

### Analytical data for 6-deoxy-6-(2-aminoethyl) aminocellulose AEA-1′

Elemental analysis: 41.60% C, 7.19% H, 8.36% N, 0% S, 4.31% Cl (experimental); 43.16% C, 5.19% H, 8.36% N, 0% S, 4.31% Cl (calculated). ^1^H NMR spectroscopy (250 MHz, D_2_O): *δ* [ppm] = 5.2–2.2 (H1–H6, H7, H10), 3.48 (H9), 2.84 (H8). ^13^C NMR (63 MHz, D_2_O) *δ* [ppm] = 102.2 (C1), 80.8 (C4), 67.0–79.5 (C2, C3, C5), 59.6, 48.5, and 47.0 (C6, C6_deoxy_), 38.8, 39.8 (C7, C8).

### Analytical data for 6-deoxy-6-(2-hydroxyethyl) aminocellulose HEA-1′

Elemental analysis: 43.59% C, 76.88% H, 4.55% N, 1.38% S, 2.31% Cl (experimental); 46.19% C, 6.64% H, 4.55% N, 1.38% S, 2.31% Cl (calculated). ^1^H NMR spectroscopy (250 MHz, D_2_O): *δ* [ppm] = 7.78–7.24 (H11 and H12), 4.5–2.5 (H1-H6, H7, H10), 3.62 (H9), 2.60 (H8).

### Sedimentation velocity in the analytical ultracentrifuge

Sedimentation coefficient distributions of aminocellulose were determined using sedimentation velocity in a Beckman XL-I analytical ultracentrifuge (AUC) equipped with Rayleigh interference optics, as previously described (Heinze et al. 2011; Nikolajski et al. 2014). 12 mm optical path double sector cells with an epoxy centrepiece and sapphire windows in an aluminium housing were employed: solution and solvent (buffer) reference channels were filled to 400 μL. A rotor speed of 50,000 rpm (~ 200,000 g) was employed for AEA-1′ and 45,000 rpm (~ 160,000 g) for HEA-1′ at a temperature of 20.0 °C. 1.0 mg/ml stock solutions of AEA-1′ and HEA-1′ were prepared in pH = 7.0, *I* = 0.10 M phosphate-chloride buffer (Green 1933) and were serially diluted. Analysis was carried out using SEDFIT (Schuck 2003; Dam and Schuck 2004) which gives an apparent distribution of (diffusion-corrected) sedimentation coefficient c*(s)* vs *s* and the corresponding (apparent) weight-average sedimentation coefficient, *s*. We use a range from 0.1 to 15 S, with regularisation (confidence F ratio) 0.95 and resolution set to 250 and the data points were fitted using B-splines. The validity of the c(*s*) approach for these materials had been examined by Heinze et al (2011) who obtained corresponding values using the least-squares g(*s*) vs *s* method (Dam and Schuck 2004) and then performing a multi-Gaussian fit (Heinze et al (2011)—Supplementary Data S3).

All sedimentation coefficients were normalised to standard conditions (density and viscosity of water at 20.0 °C)—see Tanford (1961). A value for the partial specific volume ῡ = 0.614 ml/g for AEA-1′ and ῡ = 0.619 ml/g for HEA-1′ was determined from solution and solvent density measurements using the relation of Kratky et al. (1973):

1 $$\stackrel{-}{v}=\frac{1}{{\rho }_{0}}\left(1-\frac{d\rho }{dc}\right),$$

where ρ_o_ is the density of solvent and ρ is the density of the solution at concentration *c* (g/ml).

### Sedimentation equilibrium in the analytical ultracentrifuge

The same 12 mm Beckman XL-I AUC and cells were used as above but with shorter (100 µL) solution columns and a rotor speed of 40,000 rpm (~127,000 g) and temperature of 20.0 °C for AEA-1′ and HEA-1′. Although this speed is lower than for sedimentation velocity, it is still relatively high for sedimentation equilibrium, since, as before (Nikolajski et al. 2014), we are focusing on monomer–oligomer equilibria. Loading concentrations from 0.30 to 1.0 mg/ml AEA-1′ and 0.4–1.0 mg/ml HEA-1′ in the *I* = 0.10 M phosphate chloride buffer were employed. Scans with Rayleigh interference optics were taken every hour until equilibrium was reached: this was assessed using the SEDFIT-Tools-Test approach to equilibrium (courtesy of P. Schuck). Because of the small size of the macromolecules, as before, thermodynamic non-ideality effects were assumed negligible at the concentration range studied (Nikolajski et al. 2014). MULTISIG (Gillis et al. 2013), which assumes thermodynamic ideality, was also run using its standard 17 component system with 20 iterations for each concentration yielding molar mass distributions, *M*_w_(*r*) vs *c*(*r*), *M*_n_(*r*) vs c(*r)* and *M*_z_(*r*) vs (*r)*, where *c*(*r*) is the local concentration at a radial position *r* in the cell, and n, w, and z are number, weight and z-averages for molar mass distributions respectively.

## Results

### Evidence of multiple species by sedimentation velocity

**Fig. 2:**
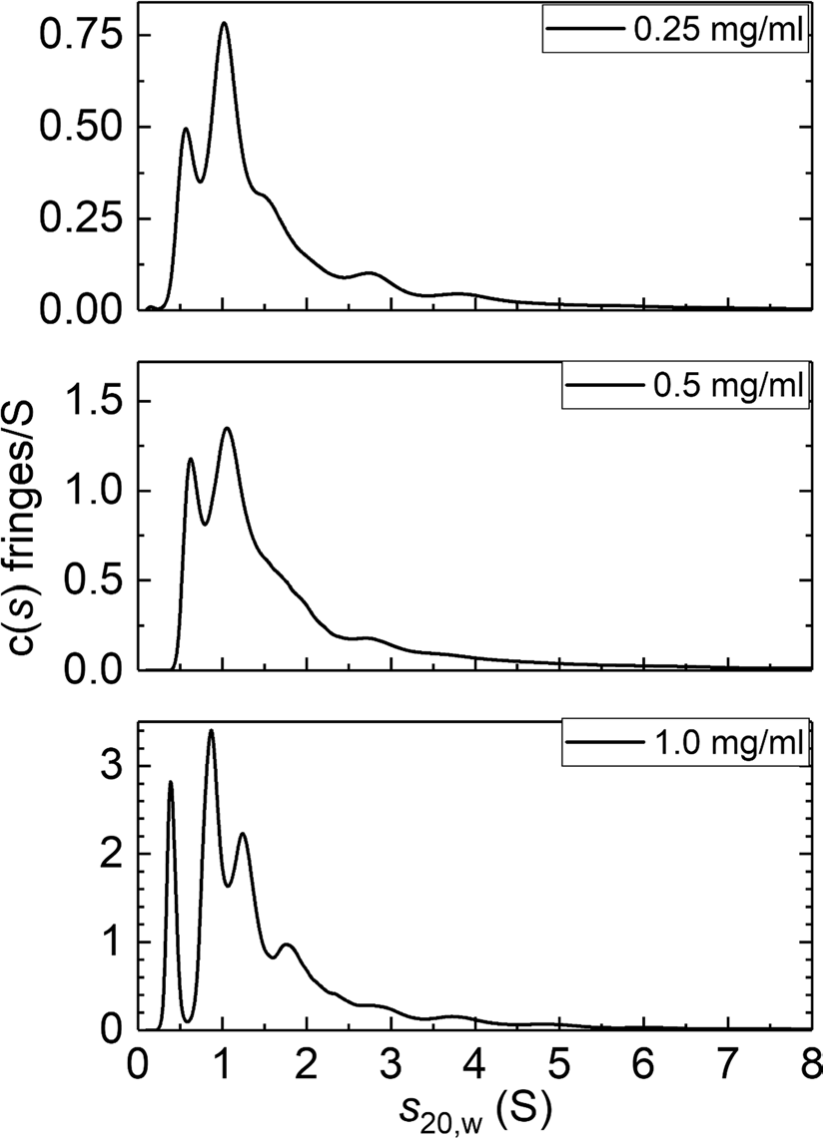
Plots of apparent, diffusion-corrected sedimentation coefficient distribution *c*(*s*) vs sedimentation coefficient *s*_20_,w (in Svedberg units, S) for aminocellulose AEA-1′ at three different loading concentrations

**Fig. 3:**
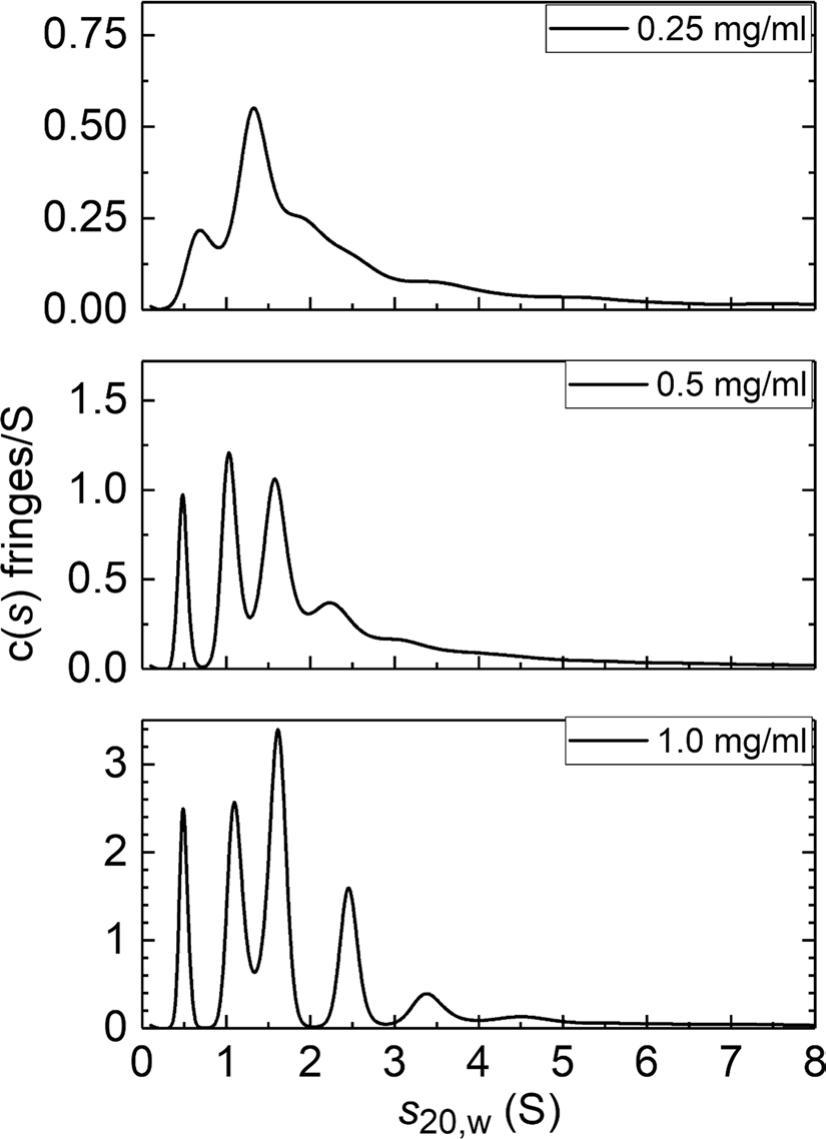
Plots of apparent, diffusion-corrected sedimentation coefficient distribution *c*(*s*) vs sedimentation coefficient *s*_20_,w for hydroxylated aminocellulose HEA-1′ at 3 different loading concentrations

Sedimentation velocity in the analytical ultracentrifuge (SV) confirmed the existence of multiple components for AEA-1′ (Fig. 2), similar to what was observed previously for AEA-1 (Heinze et al. 2011; Nikolajski et al. 2014), with > 6 species present, at the different loading concentrations ~ 0.4S, 0.9S, 1.3S, 2.0S, 3.0S, and 3.8S. Similar behaviour is seen for its hydroxylated derivative HEA-1′ (Fig. 3), again with > 6 species present at ~ 0.5S, 1.1S, 1.6S, 2.5S, 3.5S, and 5.0S. No clear increase in the proportion of the higher *s* species compared to the lower *s* species was evident, suggesting that the association of these higher species from 0.5S was only partially reversible, consistent with what was observed before. However, Nikolajski et al. (2014) found that the lowest sedimentation coefficient component (~0.5S) for AEA-1 represented a rapid reversible equilibrium: we investigate this further using high-speed sedimentation equilibrium (SE) for AEA-1′ and HEA-1’.

### Sedimentation equilibrium assay for reversibility

**Fig. 4:**
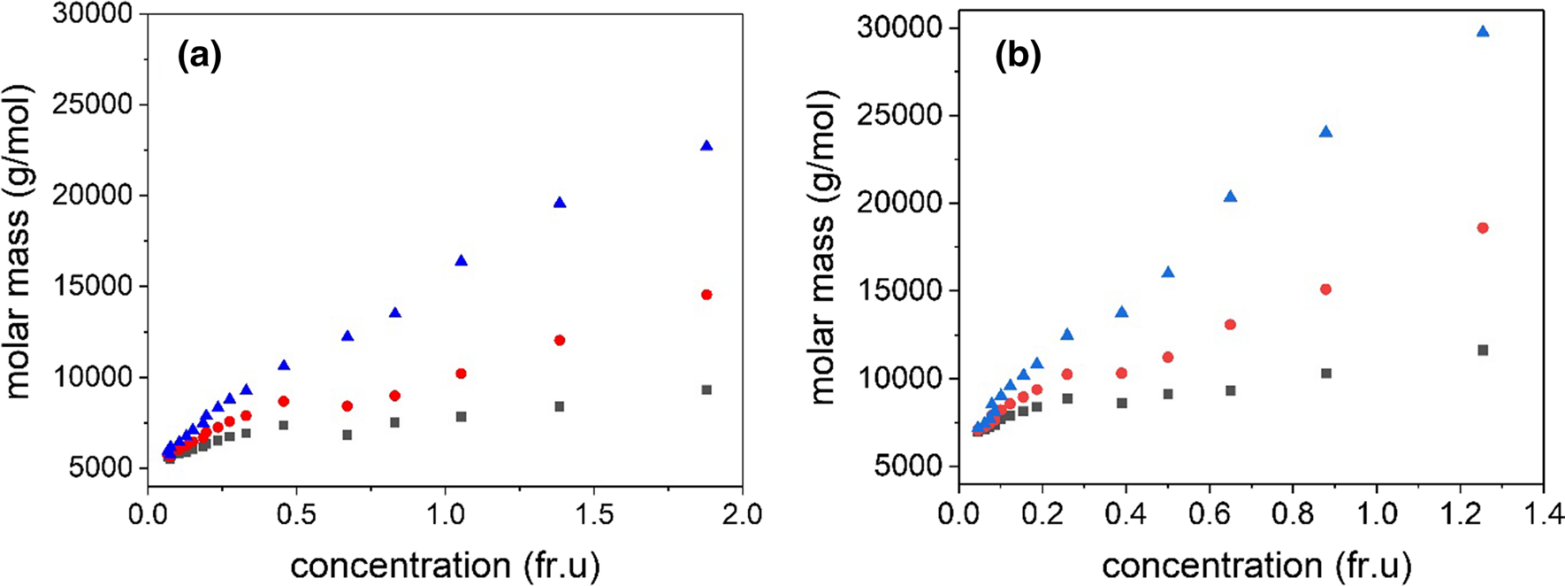
Plot of point molar mass change with concentration for **a** AEA-1′ and **b** HEA-1′ showing a self-association from sedimentation equilibrium. The plot is of the number *M*_n_(*r*), weight *M*_w_(*r*), and z-average *M*_z_(*r*) molar masses versus local concentration *J*(*r*) (fringe increment units) at radial positions *r* in the ultracentrifuge cell for an initial loading concentration *c* = 0.5 mg/ml. Black squares: n-average molar masses; red circles, weight (mass) average molar masses, blue triangles, z-average molar masses. They converge to a single value, the “monomer” *M*_1_ = 4500 g/mol for AEA-1′ as fringe concentration → 0 and *M*_1_ ~ 5500 g/mol for HEA-1’

**Fig. 5:**
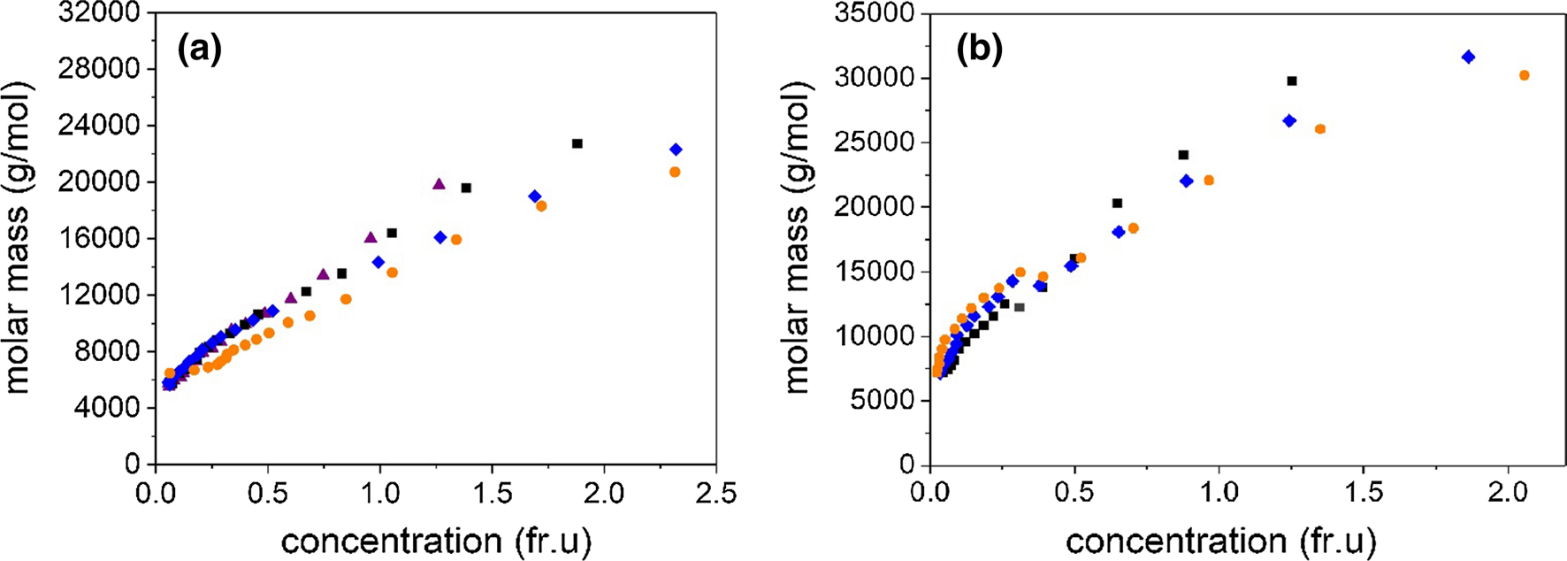
‘Overlap’ diagnostic plots from sedimentation equilibrium for a reversible self-association for AEA-1′ Plot of z-average molar masses *M*_z_(*r*) as a function of Rayleigh fringe concentration *J*(*r*) for different initial loading concentrations. Purple triangles: 0.4 mg/ml; black squares 0.5 mg/ml, blue diamonds, 0.6 mg/ml and orange circles 0.7 mg/ml initial loading concentration. **a** AEA-1′; **b** HEA-1′. The plots converge to the same values as fringe concentration → 0 for (**a**) and (**b**) as Fig. 4

We now use sedimentation equilibrium to analyse for a reversible association at lower molar masses/sedimentation coefficients for both AEA-1′ and HEA-1′ to look for similarities with what was observed before for AEA-1 (Nikolajski et al. 2014). The object—as with Nikolajski et al.—is not to get the overall weight-average molar mass over all the components but to run at higher speeds so as to focus on the smaller components and see if there is a fully reversible oligomerisation of lower molar mass species as observed before for AEA-1. We use the following assays:

(i) Convergence plots of point average molar mass plots at zero (fringe) concentration. We plot the point or local number average, weight-average, and z-average molar masses, *M*_n_(*r*), *M*_w_(*r*), and *M*_z_(*r*), respectively, as a function of local (Rayleigh fringe) concentration *J*(*r*) in the ultracentrifuge cell. The convergence of plots to a common (monomer) molar mass is indicative of a reversible self-association. As can be seen from Fig. 4, this is also the case for both AEA-1′ (Fig. 4a) and HEA-1′ (Fig. 4b). The common extrapolated value (*J*(*r*) = 0) yields monomer molar masses *M*_1_ of ~ 4500 g/mol (where 1 g/mol = 1 Da) for AEA-1′. This is slightly higher than seen previous for the related AEA-1 of 3250 g/mol. HEA-1′ gives an even larger monomer molar mass of *M*_1_ ~ 5500 g/mol.

(ii) An overlay of point average molar mass *M*_z_(*r*) vs *J*(*r*) plots for different loading concentrations *c*. The classical test of *reversibility* for a self-association is overlay of data-sets obtained at different loading concentrations of plots of point average molar masses vs *J*(*r*) (Roark and Yphantis 1969; Harding 1984; Nikolajski et al. 2014). As before (Nikolajski et al. 2014), we use the point z-average molar mass *M*_z_(*r*) in the MULTISIG algorithm which is more stable and has a higher sensitivity (Gillis et al. 2013). Figure 5a shows a plot for four loading concentrations, and good overlap is seen particularly for the lowest three 0.4, 0.5, 0.6 mg/ml up to a molar mass of ~ 12,000 g/mol. This is consistent to what has been observed before, although the range of concentrations studied here is narrower. For comparison, a good example of non-overlap for carbohydrate polymers has been given in the supplementary data (Fig S2) to Nikolajski et al (2014) for a mucin glycoprotein, together with a good example for a reversible system (Fig S1 in that paper), namely an electron transferring flavoprotein. Re-assuringly, the values extrapolate to the same value as the fringe concentration → 0, namely an *M*_1_ value of 4500 g/mol, in agreement with Fig. 4a for AEA-1′. Figure 5b shows the corresponding situation for HEA-1′. Again, there is reasonable overlap for the lower part of the molar mass range 5500–12,500 g/mol where the association is reversible. Beyond this, the association is only partially reversible. The extrapolated *M*_1_ is in agreement with Fig. 4, yielding a value of ~ 5500 g/mol. Interestingly, the association seems to approach a higher plateau value for HEA-1′ (~ 32,000 g/mol) compared with ~ 25,000 g/mol for AEA-1’.

## Discussion

This investigation has demonstrated the effects of changing side groups and degree of substitution can have on the hydrodynamic properties of a polymer. SV analysis showed that both AEA-1′ and HEA-1′ are heterogeneous samples revealing > 6 discrete species, a finding similar to those of Nikolajski et al. (2014) for the closely related aminocellulose AEA-1. However, AEA-1′ only has significant species up to 3S compared to 8S seen in AEA-1, and HEA-1′ which has species up to 4.5S. HEA-1′, therefore, appears to self-associate into comparatively higher associates. SE showed that AEA-1′ self-association is ~ fully reversible up to ~ 12 kDa similar to Nikolajski et al. (2014) who also found that AEA-1 is a fully reversible self-association up to at least 10 kDa. The difference is that the (covalent) monomeric molar mass was ~ 3250 g/mol for AEA-1, whereas, here, AEA-1′ was found to have a monomeric molar mass of ~ 4500 g/mol. HEA-1′ was found to have a monomeric molar mass of ~ 5500 g/mol. From the perspective of use as an aqueous archaeological wood-consolidation resin, these materials—based on molecular weight criteria—should be able to be infused into/ absorbed by the wood (Wakefield et al. 2018). From both the sedimentation velocity and sedimentation equilibrium experiments, it is clear that AEA-1 (from our previous study), AEA-1′ and HEA-1′ all self-associate to higher molar mass species, which although, on one hand, may limit the uptake of material and, on the other hand, might assist with curing strategies once the polymer is inside the wood.

## Concluding remarks

In the present study, we have synthesised and undertaken a biophysical characterisation of the self-associative behaviour of two novel aminocelluloses, namely 6-deoxy-6-(ω-aminoethyl) aminocellulose AEA-1′ and 6-deoxy-6-(ω-hydroxyethyl) aminocellulose HEA-1′, which possess a different degree of substitution and extended alkyl derivatisation (respectively) compared with the previously well-characterised 6-deoxy-6-(ω-aminoethyl) aminocellulose AEA-1, which typically displays a protein-like self-associative behaviour involving reversible oligomerisation followed by aggregation into larger structures. Using the powerful matrix-free techniques of sedimentation velocity (to investigate the nature and extent of association states) and sedimentation equilibrium (to assess the state of reversibility and estimate the (covalent) monomer molar masses of AEA-1′ and HEA-1′) in an analytical ultracentrifuge, it was demonstrated here that both polymers [with monomeric weight-average molar masses of 4.5 kDa (for AEA-1′) and 5.5 kDa (for HEA-1′)] possess monomer average molar masses in a range suitable for introduction into archaeological wood (see Wakefield, 2018). Both are heterogeneous in nature, with AEA-1′ possessing components up to 3S and HEA-1′ up to 4S, and both exhibit a partially reversible self-association, with AEA-1′ fully reversible up to 12 kDa. These characteristics are potentially desirable for the formulation of aqueous consolidants for use in archaeological wood as the associative behaviour into higher mass structures is expected to strengthen the wood, whilst the reversibility of AEA-1′ means that the polymer may, in principle, be removed from wood later if so desired. Further work is now underway to explore the performance and stability of archaeological or artificially aged wood treated with these materials and in comparison to the other main traditionally used “aqueous” polymer treatment, namely, polyethylene glycol—used for example in the consolidation of the Mary Rose (www.maryrose.org/conservation/) and Vasa (www.vasamuseet.se/en/research) vessels. Our aqueous soluble aminocellulose-based polymers may not be as suitable for application to alum-treated archaeological wood—such as the Oseberg artefacts (www.khm.uio.no/english/research/projects/saving-oseberg) described in Wakefield et al (2020).
